# Understanding recruitment: outcomes associated with alternate methods for seed selection in respondent driven sampling

**DOI:** 10.1186/1471-2288-13-93

**Published:** 2013-07-17

**Authors:** John L Wylie, Ann M Jolly

**Affiliations:** 1 Departments of Medical Microbiology and Community Health Sciences, University of Manitoba, Winnipeg, MB, Canada; 2 Cadham Provincial Laboratory, Manitoba Health, 750 William Ave, Winnipeg, MB R3E 3J7, Canada; 3 Centre for Communicable Diseases and Infection Control, Public Health Agency of Canada, Ottawa, ON, Canada

**Keywords:** Respondent driven sample, HIV, Sexually transmitted infection

## Abstract

**Background:**

Respondent driven sampling (RDS) was designed for sampling “hidden” populations and intended as a means of generating unbiased population estimates. Its widespread use has been accompanied by increasing scrutiny as researchers attempt to understand the extent to which the population estimates produced by RDS are, in fact, generalizable to the actual population of interest. In this study we compare two different methods of seed selection to determine whether this may influence recruitment and RDS measures.

**Methods:**

Two seed groups were established. One group was selected as per a standard RDS approach of study staff purposefully selecting a small number of individuals to initiate recruitment chains. The second group consisted of individuals self-presenting to study staff during the time of data collection. Recruitment was allowed to unfold from each group and RDS estimates were compared between the groups. A comparison of variables associated with HIV was also completed.

**Results:**

Three analytic groups were used for the majority of the analyses–RDS recruits originating from study staff-selected seeds (n = 196); self-presenting seeds (n = 118); and recruits of self-presenting seeds (n = 264). Multinomial logistic regression demonstrated significant differences between the three groups across six of ten sociodemographic and risk behaviours examined. Examination of homophily values also revealed differences in recruitment from the two seed groups (e.g. in one arm of the study sex workers and solvent users tended not to recruit others like themselves, while the opposite was true in the second arm of the study). RDS estimates of population proportions were also different between the two recruitment arms; in some cases corresponding confidence intervals between the two recruitment arms did not overlap. Further differences were revealed when comparisons of HIV prevalence were carried out.

**Conclusions:**

RDS is a cost-effective tool for data collection, however, seed selection has the potential to influence which subgroups within a population are accessed. Our findings indicate that using multiple methods for seed selection may improve access to hidden populations. Our results further highlight the need for a greater understanding of RDS to ensure appropriate, accurate and representative estimates of a population can be obtained from an RDS sample.

## Background

Populations vulnerable to HIV and other sexually transmitted and bloodborne infections (STBBI) are frequently characterized as hidden or hard-to-reach; a designation stemming from characteristics commonly associated with these populations such as homelessness or engagement in illicit behaviours. From a sampling perspective these characteristics negate the ability of researchers or public health workers to carry out traditional probability sampling methods. A common solution has been to employ various convenience sampling methods which, although clearly viable with respect to accessing these populations, are problematic in terms of generating conclusions or estimates that are generalizable to the population from which the sample was obtained.

Respondent driven sampling (RDS) was designed to overcome these issues and generate unbiased population estimates within populations thought of as hidden [1,2]. Briefly, the approach as originally described involves the selection of a small number of “seeds”; i.e. individuals who will be instructed to recruit others, with recruitment being restricted to some maximum number (typically 3 recruits maximum per person). Subsequently recruited individuals continue the process such that multiple waves of recruitment occur. Ultimately any bias associated with initial seed selection would be eliminated and the resultant sample could be used to produce reliable and valid population estimates via RDS software designed for that purpose.

The method has gained widespread acceptance over the last 15 years.; over a five year period, a 2008 review identified 123 RDS studies from 28 countries covering 5 continents and involving over 30,000 study participants [3]. However, its widespread use has been accompanied by increasing scrutiny as researchers attempt to understand the extent to which the population estimates produced by RDS are generalizable to the actual population(s) of interest. As recently noted, the “respondent-driven” nature of RDS, in which study participants carry out the sampling work, creates a situation in which data generation is largely outside the control and, potentially more importantly, the view of researchers [4].

Simulation studies and empirical assessments have been used to assess RDS results. Goel and Salganik [5] have suggested that RDS estimates are less accurate and confidence limit intervals wider than originally thought. They further note that their simulations were best-case scenarios and RDS could in fact have a poorer performance in practice than their simulations. McCreesh et al. [6] carried out a unique RDS in which the RDS sample could be compared against the characteristics of the known population from which the sample was derived. These researchers found that across 7 variables, the majority of RDS sample proportions (the observed proportions of the final RDS sample) were closer to the true population proportion than the RDS estimates (the estimated population proportions as generated by RDS software) and that many RDS confidence intervals did not contain the true population proportion. Reliability was also tested by Burt and Thiede [7] via repeat RDS samples amongst injection drug users within the same geographic area. Comparisons of several key variables suggested that materially different populations may in fact have been accessed with each round of surveying with similar results subsequently found in other studies [8,9]; although true behaviour change over time *vs*. inadvertent access of different subgroups within a larger population are not easily reconciled. The use of different sampling methods (*e.g*. RDS *vs*. time-location sampling), either done within the same area at the same time [10-12], or, less informatively, at different times and/or places [13-15], clearly demonstrate that distinct subgroups within a broader population exist and are preferentially accessed by one method over another.

The above studies demonstrate that accuracy, reliability and generalizability of RDS results are uncertain and more evaluation is required. Also, assumptions held in simulation studies may not match what occurs in reality while empirical comparisons over time or between methods do not reveal what is driving the differences in the results. Studies such as those of McCreesh et al. [6] come closest to revealing discrepancies between an RDS sample and the target population, but cannot be replicated within the “hidden” populations within which RDS is typically employed.

In this study, we conducted simultaneous, yet separate RDS studies within the same population at the same point in time which has not yet been attempted, to our knowledge. Like all of the studies described above, a study of this kind is not a definitive endpoint, but it does add to the body of RDS evaluation literature and may alert researchers of issues to be aware of when designing RDS studies. Numerous approaches are possible for designing and implementing two simultaneous RDS studies. Seeds could arbitrarily be assigned to one or the other arms of the study, or different groups of seeds could be created, with both groups generally fitting within the umbrella characteristics of the target population, but differing in some key aspect (e.g. seed groups differing by gender or age).

In this study, we compare two different methods of seed selection. One arm was initiated by creating a seed group using the typical RDS approach of study staff selecting a small number of seed individuals. The second arm was allowed to proceed in an entirely respondent-driven manner with study staff not being directly involved in either the primary seed selection or the secondary recruitment. This process is not unlike that recently used by Daniulaityte et al. [16] in which individuals who had been referred to the study but who were not in possession of a recruitment coupon were designated as seeds. Our process differed in that these alternate self-presenters were treated as a separate seed group for purposes of comparing recruitment dynamics. The individuals self-presenting to study staff could only have heard about the study through either our own staff-selected seeds or the recruits of these seeds (no other study advertisement of any kind was used), therefore, all individuals would have been in social contact with each other in some manner and hence part of a larger interconnected social network. Given this interconnectedness and social contact our hypothesis upon study initiation was that the two simultaneous RDS arms would not yield substantially different results. Any differences between the seed groups would be eliminated as recruitment unfolded and both would produce similar RDS population estimates.

## Methods

### Study implementation

Data collection took place in Winnipeg, Manitoba, Canada as part of a larger survey (Social Network Study III–SNS III) designed to better understand interactions between individuals at risk for STBBI. Based on previous experience with this study population we anticipated that word-of-mouth advertising would also occur, therefore, we used this opportunity to create the parallel RDS recruitment arms.

Questionnaire administration occurred over an 11 month period from January to December 2009. Interviewing and specimen collection was conducted by one research nurse. A variety of interview sites had been established by this nurse prior to study implementation. These interview sites were located within local clinics or resource centres geographically dispersed throughout the areas of Winnipeg where it was expected most participants would reside. Upon first phone contact with the nurse, a mutually agreed upon interview time and place was established and the nurse traveled to a given interview site at the appointed time. Similar approaches have been used by others to ensure RDS can be carried out in a cost-effective manner [17].

Each participant was paid a $40 honorarium following questionnaire administration and specimen collection. RDS coupon distribution was voluntary as no secondary incentives were provided for successful enrolment of others into the study. Three coupons were provided to study participants for purposes of recruitment. Coupons contained no expiry date and could be redeemed at any time during the data collection period.

The first arm of the study was initiated by study staff selecting seeds, as per standard RDS procedures. The research nurse selected 22 individuals. The study questionnaire was administered to each selected seed, to provide more data on the various risk groups represented by these seeds. As examples, analysis of their responses demonstrated that 15 were injection drug users (IDU); 4 were street-involved youth, 9 were sex workers, and 4 were men who have sex with men (MSM) (total exceeds 22 as some individuals were members of more than one of these groups).

The second arm of the study resulted from news of the study spreading through word of mouth within the larger social network of members of STBBI-vulnerable populations within Winnipeg. Within days of the launch of Arm 1, individuals began contacting our study nurse asking if they could be interviewed as part of the study. As noted above, given that no advertising of the study was conducted in any way by study staff, knowledge of the study was being transmitted via our study staff-selected seeds or via their initial recruits. We made no attempt to hinder enrolment of these self-presenters and accepted any of these individuals as alternate seeds for the duration of the study period. Over the duration of the study period, 118 individuals who self-presented to the study were interviewed and designated as alternate seeds.

Recruitment coupons were provided at the end of questionnaire administration. Given their familiarity with the types of questions asked, study participants were instructed to recruit other friends or family members who they believed practiced some of the risk behaviours they had been questioned about. Upon presentation, potential study participants were asked their age to meet the minimum age requirement of 14, with no other pre-screening occurring. This broad criteria was largely driven by the wide range of risk groups under investigation in the SNS III study, as one of the research aims of this larger study was to better understand bridging between different risk groups (analyses to be presented in future publications).

For the remainder of this paper, study staff-selected seeds in Arm 1 will be referred to as Arm 1 seeds, and their recruits as Arm 1 recruits. Individuals self-presenting to the study without an RDS coupon and designated as seeds are termed Arm 2 seeds and their recruits as Arm 2 recruits.

### Recruitment targets

Several considerations were taken into account in setting a target sample size. First, Johnston et al. [18] determined that the median final sample size reported from 118 RDS studies is 225 with an IQR of 152–360. As a starting point, the value of 225 was set as an approximate recruitment target within both RDS arms of the current study, such that each would generate a final sample size comparable to that typically seen in other RDS studies. Second, a formative research study in Winnipeg involving street-involved youth suggested that many individuals who self-present to a study are relatively poor recruiters (averaging approximately 2 recruits per seed) [19]. Therefore, if this recruitment pattern held in the current study, 225 recruits in arm 2 of the study would result from and be accompanied by the entry of approximately 112 self-presenting seeds. These considerations resulted in a total target sample of 562 (225 arm 1 recruits, 225 arm 2 recruits and 112 arm 2 seeds). This overall number was increased to 600 for purposes of calculating financial resources required for honoraria. Within a study of this type, different scenarios were possible with respect to allowing recruitment to proceed unhindered within the two arms *vs*. attempting to encourage/restrict recruitment within one study arm over the other if differential recruitment was clearly occurring. As noted above, in keeping with our intention to have recruitment proceed largely in a respondent-driven manner, we opted to allow recruitment to proceed unhindered within each arm and simply terminate all data collection once available honoraria resources had been depleted (600 individuals including both the 22 staff selected arm 1 seeds plus 587 additional individuals).

### Establishment of social network size

In RDS studies, in order to correct for unequal sampling probabilities, the size of each individual’s network is elicited. In RDS studies which examine very specific behaviours, precise questions regarding social network size can be constructed. In the case of this study, the broad scope of the risk behaviours of interest did not allow for simple inquiries regarding the number of network members that engaged in a specific type of behaviour. Therefore, each study participant’s egocentric social network was used as a measure of network size. Each participant was asked to list by first name, initials, or other non-identifying means, the members of their personal egocentric network. General prompts were first used to assist participants to recall their network members; individuals were asked to think of the people that they normally associate with, that are important in their lives and that they see or speak with on a regular basis. After an initial list was constructed, participants were then further prompted to think of other individuals they may have forgotten to list; here participants were asked to specifically think about their friends and family members and other people with whom they’ve had sex, used drugs together, lived, hung out or worked.

Previous experience suggested that the majority of participants would list fewer than 10 individuals within their personal networks and the questionnaire capped the network list at this number. Although individuals were allowed to indicate how many more individuals past 10 they could nominate, answers were either vague (e.g. “many more”) or were subject to lumping around specific values ending in 0 or 5, therefore, the maximum network size used in the RDS analysis was set to 10. Of the 600 people in the study, 552 (92.0%) indicated their network consisted of 10 or fewer people.

### Tracking recruitment

RDS recruitment coupons used were the size of business cards that contained the study title, “Social Network Study–SNS III”, followed by several bullet points: *“We need your help for a research study on infectious diseases; 1 hour questionnaire; diagnostic tests offered for HIV, Hepatitis C, Hepatitis B, syphilis, chlamydia, gonorrhea; Honorarium provided; Please phone xxx-xxxx if you are interested in taking part and ask for the research nurse; please give this coupon to the research nurse when you see her*”. On the back of each card, and with the participant’s knowledge, individual study codes corresponding to the interviewed person were written on each of three cards given to a participant; individual cards were distinguished using 01, 02, or 03 as a suffix to the study code. The individual study codes and suffixes were used to establish recruitment chains for the RDS.

### Questionnaire measures

The questionnaire was designed to investigate several aspects of substance use and sexual behaviours of study participants. We used a subset of key sociodemographic and behavioural variables to compare RDS recruitment across the two RDS Arms (Table 1). Self reported gender was categorized as male, female or transgender. Ethnicity consisted of Caucasian, First Nation, Métis and other/unsure (First Nation was inclusive of all Aboriginal groups with the exception of Métis; this latter group consists of individuals of mixed European and First Nations ancestry). Main income from part or full time employment was differentiated from monetary support from friends, family, government (e.g. welfare or employment insurance) or various types of illegal income. Housing was coded as “private residence” where individuals lived in an apartment or house belonging to the participant, a friend, or family member, while “public housing” consisted of unstable housing such as shelters, hotels, boarding houses, or on the street.

**Table 1:** Characteristics of study participants by type of recruitment

***Variable***	**Arm 1 recruits (n = 196)**	**Arm 2 seeds (n = 118)**	**Arm 2 recruits (n = 264)**	***p*value**
**Education**				
*Graduate or in school*	84 (42.9)	29 (24.6)	96 (36.3)	***0.005***
*Dropped out or unsure*	112 (57.1)	89 (75.4)	168 (63.6)	
**Income**				
*Full/part-time work*	36 (18.4)	8 (6.8)	39 (14.8)	***0.017***
*Support*	160 (81.6)	110 (93.2)	225 (85.2)	
**Housing**				
*Private residence*	106 (54.1)	54 (45.8)	160 (60.6)	***0.024***
*Public residence*	90 (45.9)	64 (54.2)	104 (39.4)	
**Gender**				
*Male*	110 (56.1)	53 (44.9)	143 (54.2)	0.191
*Female*	82 (41.8)	64 (54.2)	119 (45.1)	
*Transgender*	4 (2.0)	1 (0.9)	2 (0.8)	
**Ethnicity**				
*Caucasian*	62 (31.6)	10 (8.5)	57 (21.6)	***<0.0001***
*Aboriginal*	80 (40.8)	80 (67.8)	150 (56.8)	
*Metis*	42 (21.4)	24 (20.3)	47 (17.8)	
*Other/unsure*	12 (6.1)	4 (3.4)	10 (3.8)	
**Solvent use**				
*No*	136 (69.4)	64 (54.2)	147 (55.7)	***0.004***
*Yes*	60 (30.6)	54 (45.8)	117 (44.3)	
**IDU**				
*No*	101 (51.5)	53 (44.9)	136 (51.5)	0.441
*Yes*	95 (48.8)	65 (55.1)	128 (48.5)	
**Street-involved youth**				
*No*	161 (82.1)	99 (83.9)	246 (93.2)	***0.001***
*Yes*	35 (17.9)	19 (16.1)	18 (6.8)	
**MSM**				
*No*	179 (91.3)	115 (97.5)	254 (96.2)	***0.023***
*Yes*	17 (8.7)	3 (2.5)	10 (3.8)	
**Sex work**				
*No*	182 (92.9)	97 (82.2)	228 (86.4)	***0.014***
*Yes*	14 (7.1)	21 (17.8)	36 (13.6)	

IDU were those who had ever injected non-prescription drugs; solvent users were those who had ever sniffed any solvents (solvent use was a focus of our larger study and was included here to inform future analyses). Street-involved youth were 14–24 years and further reported having “ever taken off or run away from home for 3 or more consecutive nights”. A series of questions were used to elicit MSM and sex work behaviours from study participants. Sex work included “survival sex” and was defined as being provided with money, drugs, food, clothes or shelter in exchange for sex.

### Biological specimens

Individuals consenting to serum testing for HIV were offered a follow-up appointment to receive their results and assistance with accessing appropriate health care. HIV testing was conducted using the ADVIA Centaur® HIV 1/0/2 Assay HIV (Siemens). All testing was carried out at Cadham Provincial Laboratory, Winnipeg, Manitoba, Canada. Of the 600 people in the study, 508 (84.7%) provided a serum specimen.

### Sample analysis

Data analysis focused on a comparison of the sample groups obtained via the separate RDS arms. Pajek [20] was used to identify the number and size of individual recruitment chains. The analysis summarized in Table 1 used Chi square analysis to identify overall differences between the arm 1 recruits, the arm 2 seeds, and the arm 2 recruits. The analysis of Table 2 used multinomial logistic regression to identify differences between the arm 1 recruits (used as the reference group) and the arm 2 seeds or arm 2 recruits. The 22 arm 1 seeds were not included, given their small number and purposeful selection. In the multinomial analysis, the effect of removing variables was assessed through the likelihood ratio test.

**Table 2:** Final multivariable multinomial logistic regression model of outcome measures associated with recruitment type

	**Arm 2 seeds**		**Arm 2 recruits**	
	**OR (95% CI)**	**p value**	**OR (95% CI)**	**p value**
**Education**				
*Dropped out or unsure*	**1.83 (1.09, 3.11)**	**0.023**	1.16 (0.78, 1.72)	0.475
**Income**				
*Support*	**2.47 (1.09, 3.11)**	**0.031**	1.13 (0.67, 1.91)	0.635
**Housing**				
*Public residence*	1.23 (0.77, 1.99)	0.385	0.73 (0.49, 1.07)	0.106
**Solvent use**				
*Yes*	1.62 (0.99, 2.67)	0.056	**1.60 (1.06, 2.40)**	**0.023**
**Street-involved youth**				
*Yes*	0.85 (0.45, 1.62)	0.621	**0.34 (0.18, 0.63)**	**0.001**
**MSM**				
*Yes*	**0.24 (0.06, 0.89)**	**0.033**	**0.33 (0.14, 0.78)**	**0.012**
**Sex work**				
*Yes*	**2.59 (1.21, 5.50)**	**0.013**	**2.36 (1.19, 4.67)**	**0.013**

Significant differences are referenced against Arm 1 recruits.

The analysis for Table 3 used RDSAT version 5.6 [21] to generate the RDS measures of estimated population proportion and homophily. Homophily values in RDS can vary from -1.0 to 1.0. Values near 0 indicate random recruitment (e.g. a value of 0 for individuals with male gender would indicate that males were equally likely to recruit a male participant as a female participant). Positive homophily values indicate a tendency to recruit others who share a given characteristic, while the opposite is true for negative values.

**Table 3:** Comparison of seed and recruit sample sizes (and proportions for recruits), homophily and estimated population proportions between arms 1 and 2

	**Arm 1**				**Arm 2**			
***Variable***	**Seeds**	**Recruits (observed proportion)**	**Estimated population proportion (CI)**	**Homophily**	**Seeds**	**Recruits (observed proportion)**	**Estimated population proportion (CI)**	**Homophily**
**Education**								
*Graduate or in school*	6	84 (0.43)	0.39 (0.32-0.45)	0.086	29	96 (0.36)	0.32 (0.26-0.37)	0.144
*Dropped out or unsure*	17	111 (0.57)	0.61 (0.55-0.68)	-0.061	89	168 (0.64)	0.69 (0.63-0.74)	-0.037
**Income**								
*Full/part-time work*	5	36 (0.19)	0.17 (0.12-0.23)	0.093	8	39 (0.15)	0.15 (0.10-0.20)	0.026
*Support*	18	159 (0.82)	0.83 (0.77-0.88)	0.021	110	225 (0.85)	0.85 (0.80-0.90)	0.037
**Housing**								
*Private residence*	11	106 (0.54)	0.52 (0.44-0.6)	***0.326***	54	160 (0.61)	0.64 (0.55-0.72)	***0.482***
*Public residence*	12	89 (0.46)	0.48 (0.4-0.56)	0.205	64	104 (0.40)	0.37 (0.29-0.45)	***0.345***
**Gender**								
*Male*	12	109(0.56)	0.61 (0.54-0.67)	-0.011	53	143 (0.54)	0.55 (0.48-0.63)	0.104
*Female*	8	82(0.42)	0.38 (0.31-0.45)	0.153	64	119 (0.46)	0.45 (0.37-0.52)	0.11
*Transgender*	3	4 (0.02)	0.02 (0.004-0.03)	-1.0	1	2 (0.01)	0.004 (0.002-0.01)	-1.0
**Ethnicity**								
*Caucasian*	5	62 (0.32)	0.32 (0.24-0.39)	0.113	10	57 (0.22)	0.28 (0.21-0.35)	0.096
*Aboriginal*	13	79 (0.41)	0.42 (0.32-0.51)	***0.302***	80	150 (0.57)	0.46 (0.38-0.55)	***0.409***
*Metis*	2	42(0.22)	0.19 (0.14-0.24)	0.102	24	47 (0.18)	0.20 (0.15-0.27)	0.013
*Other and unsure*	3	12(0.06)	0.074 (0.03-0.13)	0.1	4	10 (0.04)	0.06 (0.03-0.09)	-1.0
**Solvent use**								
*No*	11	135 (0.70)	0.70 (0.64-0.75)	-0.024	64	147 (0.56)	0.58 (0.5-0.65)	0.238
*Yes*	12	60(0.31)	0.30 (0.25-0.36)	-0.083	54	117 (0.44)	0.43 (0.35-0.50)	0.224
**IDU**								
*No*	8	100 (0.51)	0.56 (0.47-0.64)	0.28	53	136 (0.52)	0.50 (0.42-0.56)	0.187
*Yes*	15	95 (0.49)	0.44 (0.35-0.53)	***0.345***	65	128 (0.49)	0.5 (0.44-0.58)	0.072
**Street-involved youth**								
*No*	18	161 (0.83)	0.84 (0.79-0.89)	0.129	99	246 (0.93)	0.95 (0.93-0.98)	-0.011
*Yes*	5	34(0.17)	0.16 (0.11-0.22)	0.262	19	18 (0.07)	0.05 (0.03-0.07)	0.141
**MSM**								
*No*	19	178(0.91)	0.90 (0.86-0.94)	0.072	115	254 (0.96)	0.97 (0.95-0.99)	-0.008
*Yes*	4	17 (0.09)	0.10 (0.06-0.14)	***-0.351***	3	10 (0.04)	0.03 (0.01-0.05)	***-1.0***
**Sex work**								
*No*	14	181(0.93)	0.94 (0.92-0.97)	-0.019	97	228 (0.86)	0.87 (0.83-0.91)	0.068
*Yes*	9	14 (0.07)	0.06 (0.03-0.09)	-0.202	21	36 (0.14)	0.13 (0.09-0.18)	0.103

For homophily, values exceeding 0.3 or -0.3 are in bold.

Analyses of Tables 4 and 5 used Fisher’s exact test and exact logistic regression [22] to assess associations between HIV and the outcome measures within each recruitment arm. Exact statistics were used due to low cell sizes. Similar to Rudolph et al. [11], we applied no RDS weights to any analysis as our analyses were meant to only compare the sample groups recruited within the two arms. All regression analyses were carried out in Stata version 11.1 (Stata Corporation, College Station, TX).

**Table 4:** Comparisons of outcome measures associated with HIV by each type of recruitment. Outcome measures showing significant differences by Fisher’s Exact test are indicated in bold font

	**Arm 1 recruits**			**Arm 2 seeds**			**Arm 2 recruits**		
	**HIV**			**HIV**			**HIV**		
	**Negative**	**Positive**		**Negative**	**Positive**		**Negative**	**Positive**	
	**(n = 158)**	**(n = 16)**		**(n = 81)**	**(n = 18)**		**(n = 223)**	**(n = 12)**	
**Education**									
*Graduate or in school*	66 (41.8)	6 (37.5)		22 (27.2)	2 (11.1)		**83 (37.2)**	**0 (0.0)**	**
*Dropped out or unsure*	92 (58.2)	10 (62.5)		59 (72.8)	16 (88.9)		**140 (62.8)**	**12 (100.0)**	
**Income**									
*Full/part-time work*	32 (20.3)	1 (6.3)		6 (7.4)	0 (0.0)		36 (16.1)	1 (8.3)	
*Support*	126 (79.8)	15 (93.8)		75 (92.6)	18 (100.0)		187 (83.9)	11 (91.7)	
**Housing**									
*Private residence*	90 (57.0)	6 (37.5)		37 (45.7)	7 (38.9)		137 (61.4)	4 (33.3)	
*Public residence*	68 (43.0)	10 (62.5)		44 (54.3)	11 (61.1)		86 (38.6)	8 (66.7)	
**Gender**									
*Male*	87 (55.1)	10 (62.5)		34 (42.0)	8 (44.4)		125 (56.1)	8 (66.7)	
*Female*	67 (42.4)	6 (37.5)		47 (58.0)	10 (55.6)		97 (43.5)	4 (33.3)	
*Transgender*	4 (2.5)	0 (0.00)		0 (0.0)	0 (0.0)		1 (0.5)	0 (0.0)	
**Ethnicity**									
*Caucasian*	52 (32.9)	4 (25.0)		6 (7.4)	1 (5.6)		50 (22.4)	1 (8.3)	
*Aboriginal*	58 (36.7)	9 (56.3)		53 (65.4)	12 (66.7)		125 (56.1)	11 (91.7)	
*Metis*	38 (24.1)	2 (12.5)		19 (23.5)	4 (22.2)		41 (18.4)	0 (0.0)	
*Other/unsure*	10 (6.3)	1 (6.3)		3 (3.7)	1 (5.6)		7 (3.1)	0 (0.0)	
**Solvent use**									
*No*	109 (69.9)	7 (43.8)		45 (55.6)	7 (38.9)		**127 (57.0)**	**3 (25.0)**	*
*Yes*	49 (31.0)	9 (56.3)		36 (44.4)	11 (61.1)		**96 (43.1)**	**9 (75.0)**	
**IDU**									
*No*	87 (55.1)	5 (31.3)		**40 (49.4)**	**2 (11.1)**	**	**122 (54.7)**	**1 (8.3)**	**
*Yes*	71 (44.9)	11 (68.8)		**41 (50.6)**	**16 (88.9)**		**101 (45.3)**	**11 (91.7)**	
**Street-involved youth**									
*No*	129 (81.6)	16 (100.0)		68 (84.0)	17 (94.4)		207 (92.8)	12 (100.0)	
*Yes*	29 (18.4)	0 (0.0)		13 (16.1)	1 (5.6)		16 (7.2)	0 (0.0)	
**MSM**									
*No*	**147 (93.0)**	**11 (68.8)**	**	78 (96.3)	18 (100.0)		213 (95.5)	12 (100.0)	
*Yes*	**11 (7.0)**	**5 (31.3)**		3 (3.7)	0 (0.0)		10 (4.5)	0 (0.0)	
**Sex work**									
*No*	151 (95.6)	14 (87.5)		67 (82.7)	13 (72.2)		190 (85.2)	12 (100.0)	
*Yes*	7 (4.4)	2 (12.5)		14 (17.3)	5 (27.8)		33 (14.8)	0 (0.0)	

Arm 1 recruits, arm 2 seeds, and arm 2 recruits have each been analyzed separately.

*p < 0.05.

**p < 0.01.

**Table 5:** Final exact logistic regression models of outcome measures associated with HIV for each type of recruitment

	**OR (95% CI)**	**p value**
**Arm 1 recruits**		
MSM		
*Yes*	**5.97 (1.38, 23.27)**	**0.0163**
**Arm 2 seeds**		
IDU		
*Yes*	**7.67 (1.63, 73.08)**	**0.0045**
**Arm 2 recruits**		
Education		
*Dropped out or unsure*	**7.37 (1.16, +inf)**	**0.0309**
Solvent use		
*Yes*	1.85 (0.40, 11.91)	0.6013
IDU		
*Yes*	7.92 (0.97, 374.19)	0.0553

### Ethics

Identifying information was not recorded as part of questionnaire data and all testing of biological specimens was by anonymous code linked to the questionnaire. The study was approved by the Health Research Ethics Board of the University of Manitoba.

## Results

### Recruitment summary

Seventeen (77.3%) of the 22 study staff selected seeds in Arm 1 successfully recruited other individuals to the study. These seeds recruited a total of 196 study participants (mean recruitment of 8.9 per seed). The largest recruitment chain within arm 1 consisted of 45 people (not including the seed). The mean number of recruits per RDS chain within arm 1 was 11.5, with 6 chains containing 10 or more individuals. For these latter 6 chains, the number of waves of recruitment ranged from 5–9, with a mean of 7.

Within arm 2, 118 individuals self presented to study staff and were designated as Arm 2 seeds. Of these, 108 agreed to attempt recruitment with 63 successfully recruiting. At close of data collection, arm 2 recruits numbered 264 resulting in a mean recruitment of 2.2 individuals per arm 2 seed. The largest recruitment chain consisted of 34 individuals. The mean number of recruits per chain within arm 2 was 4.2, with 6 chains containing 10 or more individuals. For these latter 6 chains, the number of waves of recruitment ranged from 4–6, with a mean of 5.

### Logistic regression modeling of RDS arms

Univariable analysis of the ten sociodemographic and risk behaviour outcome measures chosen for analysis, demonstrated that the majority showed significant differences at the p < 0.05 level (education, income, housing, ethnicity, solvent use, street-involved youth, MSM, and sex work) (Table 1). In the final multinomial model (Table 2), seven outcome measures remained (although insignificant, housing was included due to better fit). With arm 1 recruits as the reference group, both arm 2 seeds and recruits were more likely to be sex workers (odds ratios [OR] of 2.59 and 2.36, respectively) and less likely to be MSM (OR of 0.24 and 0.33, respectively). Arm 2 seeds only were more likely to have dropped out of school (OR of 1.83) and to have income from non-employment sources (OR of 2.47) while arm 2 recruits were more likely to be solvent users (OR of 1.60; arm 2 seeds approached significance for this variable with a *p* of 0.056 and OR of 1.62)) and less likely to be street-involved youth (OR of 0.34).

### RDS measures

RDS measures for all outcome variables are shown in Table 3. Homophily values in both arms varied widely ranging from -1.0 in some instances to 0.482 for individuals reporting their housing status as private residence. A comparison of homophily values between arm 1 and arm 2 demonstrates that some values were consistent between the two RDS arms (all values shown in parentheses below are homophily values). As an example, individuals in both arm 1 and 2 who reported living in a private residence were more likely to recruit others who lived in private residences (0.326 and 0.482, respectively). Other variables showed distinct differences between the arms. In arm 1, sex workers and solvent users tended not to recruit other sex workers or solvent users (-0.202 and -0.083, respectively) while the opposite was true for arm 2 sex workers and solvent users (0.103 and 0.224, respectively). In some situations the direction of recruitment was the same but of a different magnitude. While MSM in arm 1 showed a tendency to not recruit other MSM (-0.351), this trend was most pronounced in arm 2, where none of the MSM participants recruited other MSM (-1.0).

Discrepancies between the two arms were further accentuated by a comparison of the corresponding estimated population proportions and confidence intervals (Table 3). Arm 1 and arm 2 confidence limits for four variables either did not overlap or overlapped only by 0.01 (the latter for the solvent use variable). The population proportions estimated for solvent users and sex workers were higher in arm 2 than in arm 1 (0.43 for solvent users in arm 2 vs. 0.30 in arm 1 and 0.13 for sex work in arm 2 vs. 0.06 in arm 1). For street-involved youth and MSM, the opposite was true with population proportions in arm 1 higher than in arm 2 (0.16 for street-involved youth in arm 1 vs. 0.05 in arm 2 and 0.10 for MSM in arm 1 vs. 0.03 in arm 2).

### HIV as an outcome variable

Given that many RDS studies focus on the associations between STBBI and the characteristics of populations vulnerable to these infections, we examined the extent to which our chosen outcome measures were associated with HIV. Arm 1 recruits, arm 2 seeds and arm 2 recruits were treated as separate groups. Due to relatively small sample sizes within groups and some 0 cells, we used Fisher’s exact test for univariable analysis and exact logistic regression for multivariable analysis.

At the univariable level, HIV was associated only with MSM in arm 1 recruits; in arm 2 seeds HIV was associated with IDU; in arm 2 recruits, HIV was associated with education, solvent use, and IDU (Table 4). Exact logistic regression produced OR of 5.97 for MSM in arm 1 recruits and 7.67 for IDU in arm 2 seeds, respectively (Table 5). Exact logistic regression indicated only education as significantly associated with HIV with an OR of 7.37 in arm 2 recruits although IDU approached significance with a p value of 0.0553 and an OR of 7.92.

## Discussion

In this study we describe the results obtained when a different seed selection process was used to obtain two RDS samples within the same study setting over the same period of time. In addition to the standard RDS process of study staff specifically selecting seeds to initiate recruitment chains, we used the phenomenon of word-of-mouth advertising within a study population to designate individuals who self-select to a study as an alternate seed group. Given that word of the study could only have originated from our original seeds (and/or their recruits), all study participants would, in some manner, be part of the same social network in which messaging regarding the study is occurring. Our initial assumption and generation of hypotheses prior to study initiation was that this continuity would result in relatively similar samples being generated within the two arms of the study. In contrast, we identified numerous differences between the two arms with respect to our chosen outcome measures. We found that these differences were further manifested by the differing associations that occurred between HIV and the various analytic groups that we were able to create.

In general we found that the individuals that self-presented and became arm 2 seeds were relatively poor recruiters with an average of 2.4 recruits per seed *vs*. 8.9 in the staff selected arm 1 seeds. However, this poor recruitment was not universal for all arm 2 seeds, as the number of large recruitment chains was similar between the two arms. The individuals in Arm 2, in particular the arm 2 seeds, may represent the most marginalized members of the overall population from which we were sampling (for example, based on their lower education and income levels and greater likelihood of being solvent users–see Table 2). This marginalization may be one of the underlying determinants that governed their apparent lesser likelihood of obtaining an RDS coupon from any of the individuals in Arm 1. This occurred despite their apparent social connection to the population (i.e. without any advertising they still became aware of the study and obtained sufficient study information to initiate contact with the study nurse). Our data does not reveal whether this potential exclusion would have been inadvertent or purposeful on the part of the individuals enrolled in Arm 1, but it does raise questions as to whether the most marginalized members of a target population may be the least likely to have the means to enter a typical RDS study. Marginalization and enrolment in studies of this kind is an area that deserves further research to ensure the most marginalized and vulnerable members of a population are not inadvertently being excluded from enrolment and hence essentially remaining unknown to study staff.

With respect to specific risk groups, the two arms clearly did differ in terms of their final relative proportions. Compared to arm 1 recruits, arm 2 seeds comprised more sex workers and solvent users, who tended to recruit people like themselves. Conversely, MSM were more common amongst arm 1 recruits than either arm 2 seeds or their recruits. Individuals who had dropped out of school or who depended on non-employment sources of income were initially overrepresented amongst arm 2 seeds, but recruitment within this arm did not maintain this difference as arm 2 recruits tended to converge towards the proportions seen in arm 1. Finally, the proportion of street-involved youth was similar between arm 2 seeds and arm 1 recruits, however, arm 2 recruits ultimately diverged to a lower proportion.

Differences between the two arms persisted in comparisons of variables associated with HIV. HIV was more frequently identified within MSM amongst arm 1 recruits while it tended to be associated with education status and IDU within arm 2. Notably, IDU was not a variable that emerged as being proportionately different between arm 1 and 2, suggesting that more subtle differences occurred within the two arms that was not immediately apparent in our initial assessment of outcome measures.

These differences did not originate due to differential omission or inclusion of specific subgroups within the two seed groups; rather differential recruitment appears to have driven the samples towards their final endpoints. As noted above, arm 1 and arm 2 samples diverged to such an extent that confidence intervals for some proportions in the two groups failed to overlap. Mutually exclusive confidence intervals have been found in other RDS studies that included repeat sampling over time [7]. Our similar findings using data collected at the same point in time indicate the need for continued evaluation of RDS and the extent to which these differences are due only to the methodology itself.

Our study design has several limitations: 1) By simultaneously having two RDS comparison arms operating, it is impossible to know what results would have been obtained if we had only conducted a standard RDS recruitment study on its own. In a standard RDS study, only individuals presenting with coupons would have been eligible to enrol and we cannot ascertain whether some or many of the individuals who were, in reality, enrolled in arm 2 would have eventually received a coupon from an arm 1 individual and entered the study. This in itself may not necessarily have improved the estimates nor resulted in a simple blending of the two arms as different subgroups could have been over- or under-represented in any alternate scenario; 2) The existence of two study arms could have introduced some bias in recruitment if participants were aware of this aspect of the study. However, in this study, the existence of two study arms should not have had any influence on the study participants as the RDS coupons were not marked in any way that would identify which arm a coupon belonged to; 3) With respect to methods for creating distinct seed groups, as noted in the introduction, numerous options are possible and different results may have been obtained if a different process had been chosen; 4) Study eligibility criteria and the stringency of those criteria could also influence results; 5) In the present study, although we identified differences between the two arms, the lack of known population data, negates our ability to know which if any of the two arms produced the best population estimates. This is a problem that hinders most empirical assessments amongst hidden populations. Further, in our case we have no other contemporaneous cross-sectional surveys available that would allow us to compare our results to other, independently gathered results in this area; 6) Our egocentric network measure that was used as an input for the RDS software differs somewhat from the typically much narrower type of risk behaviour network measure used in most RDS studies. This was necessary given the broad range of risk groups that were a part of this study and could affect some RDS measures such as the estimated population proportions. However, the majority of results presented in this paper (i.e. Tables 1, 2, 4 and 5) would not be affected by this network size data; 7) the number of waves of recruitment seen in some RDS studies exceeds the maximum number of waves we obtained (9 waves in one of the Arm 1 recruitment chains) and it is possible that eventually recruitment differentials of the type we observed would diminish if a sufficiently large number of waves can be completed. Future studies can be designed to address this question; 8) our recruitment involved very broad risk groups whereas the majority of RDS studies typically have narrower recruitment criteria, and, as noted above, recruitment differentials may have eventually diminished in our sample. Overall, the criteria for enrolment and recruitment in published RDS studies do vary depending on the research question. Given this variation it would be important to understand what effect enrolment criteria has on the number of waves of recruitment that may be required in different scenarios.

## Conclusions

RDS is clearly valuable as a cost-effective data collection tool for hidden populations, especially in circumstances where researchers themselves may have limited means or knowledge to access those populations. We have demonstrated that self presenting seeds who meet eligibility criteria and those selected by knowledgeable field workers in the same study period can produce different RDS results. While all of these individuals likely belong to a larger network through which information on our study diffused, we believe we accessed different subgroups within the larger population. This method of allowing self-presenting seeds to participate and recruit increased the variation in the sample beyond staff chosen seeds. In this way, the self presenting seeds and their recruits have revealed more of the entire network of vulnerable people which can only improve our abilities to estimate risk. Our results and those of others indicate that a greater understanding of RDS methodology is necessary to ensure appropriate, accurate and representative estimates of a population can be obtained from an RDS sample. Future analyses of our data set are intended to better understand the underlying patterns in recruitment that may have contributed to the results we obtained and potentially aid in the design of RDS studies.

## Competing interests

The authors declare that they have no competing interests.

## Authors’ contributions

JLW and AMJ both contributed equally to the design of the study and obtaining financial support. JLW drafted the manuscript and performed the statistical analysis. Both authors have read and approved the final manuscript.

## Pre-publication history

The pre-publication history for this paper can be accessed here:

http://www.biomedcentral.com/1471-2288/13/93/prepub
